# Deciphering the relationship among phosphate dynamics, electron-dense body and lipid accumulation in the green alga *Parachlorella kessleri*

**DOI:** 10.1038/srep25731

**Published:** 2016-05-16

**Authors:** Shuhei Ota, Mai Yoshihara, Tomokazu Yamazaki, Tsuyoshi Takeshita, Aiko Hirata, Mami Konomi, Kenshiro Oshima, Masahira Hattori, Kateřina Bišová, Vilém Zachleder, Shigeyuki Kawano

**Affiliations:** 1 Department of Integrated Biosciences, Graduate School of Frontier Sciences, University of Tokyo, Kashiwa, Chiba, 277-8562, Japan; 2 CREST, Japan Science and Technology Agency, Tokyo, Japan; 3 Bioimaging Center, Graduate School of Frontier Science, University of Tokyo, Kashiwa, Chiba, 277-8562, Japan; 4 Hitachi High-Technologies Corporation, Science & Medical Systems Business Group, Nishi-shinbashi, Tokyo, 105-8717, Japan; 5 Center for Omics and Bioinformatics, Graduate School of Frontier Sciences, University of Tokyo, Kashiwa, Chiba, 277-8561, Japan; 6 Institute of Microbiology, CAS, Centre Algatech, Laboratory of Cell Cycles of Algae, Třeboň, Czech Republic

## Abstract

Phosphorus is an essential element for life on earth and is also important for modern agriculture, which is dependent on inorganic fertilizers from phosphate rock. Polyphosphate is a biological polymer of phosphate residues, which is accumulated in organisms during the biological wastewater treatment process to enhance biological phosphorus removal. Here, we investigated the relationship between polyphosphate accumulation and electron-dense bodies in the green alga *Parachlorella kessleri*. Under sulfur-depleted conditions, in which some symporter genes were upregulated, while others were downregulated, total phosphate accumulation increased in the early stage of culture compared to that under sulfur-replete conditions. The P signal was detected only in dense bodies by energy dispersive X-ray analysis. Transmission electron microscopy revealed marked ultrastructural variations in dense bodies with and without polyphosphate. Our findings suggest that the dense body is a site of polyphosphate accumulation, and *P. kessleri* has potential as a phosphate**-**accumulating organism.

Phosphorus (P) is a non-renewable resource and is an essential element for life on earth, as well as for modern food production. Applications of inorganic fertilizers were expanded after the 1950s, and approximately 17.6 M tons (Mt) of P were extracted worldwide from finite phosphate rock for conversion to fertilizers in 2009^1^,^2^. Several studies have reported that maximum phosphorus production could be reached in the near future. Cordell and White^3^ reviewed literature on the scarcity of global phosphorus and sustainable future pathways and discussed key issues for global phosphorus security. Eutrophication of surface water in aquatic ecosystems is another phosphate-related environmental issue. Some studies have focused on phosphorus removal in waste stabilization ponds^4^,^5^. Some algae can store phosphate (Pi) through luxury uptake of Pi, and the Pi enriched biomass has a potential for using as algae-based bio-fertilizer^6^.

Poly-P is a biological polymer composed of inorganic phosphate (Pi) residues linked by “high-energy” phosphoanhydride bonds^7^,^8^. Poly-P has a wide range of regulatory functions, including roles as energy and inorganic phosphate reservoirs^7^,^8^, and is found in prokaryotes^9^,^10^,^11^, eukaryotes^12^,^13^,^14^,^15^,^16^,^17^, and unicellular algae, such as *Cyanidioschyzon merolae*^18^,^19^, *Chlamydomonas reinhardtii*^20^,^21^, and *Chlorella* species^22^,^23^. The well-known fluorescent label for nucleic acids, 4′,6-diamidino-2-phenylindole (DAPI), interacts with poly-P at high concentrations to form DAPI–poly-P complexes that allow poly-P-rich granules to be visualized by fluorescence microscopy at an emission wavelength of 475–525 nm^24^, which produces a yellowish color. This DAPI-staining method is commonly used to detect poly-P-rich organelles *in vivo* and *in vitro*^16^,^25^,^26^.

Macronutrient (nitrogen, phosphate, and sulfur) limitation is a trigger for enhancing lipid accumulation in microalgae^27^. Some studies have used nitrogen deprivation, while others have focused on sulfur (S) deprivation, which can induce starch accumulation^27^,^28^. The relationship between nutrient limitation and polyphosphate accumulation has been the subject of several reports^15^,^21^,^29^,^30^. However, the relationship between sulfur deprivation and polyphosphate accumulation remains unclear. In this study, we revealed the relationship between DBs and poly-P dynamics under sulfur-deficient conditions in *Parachlorella kessleri*. *P. kessleri*, formerly known as *Chlorella kessleri*, is a freshwater green microalga in the family Chlorellaceae and class Trebouxiophyceae^31^. Because algal biomass and lipid studies have been performed on *P. kessleri*, it is a representative species for research into algal biofuel and biomass applications^27^,^32^,^33^,^34^. Although the association between lipid accumulation and S-deficiency has been reported^32^,^33^, the relationship between Pi dynamics and nutrient limitation remains unknown, and the site of poly-P accumulation in S-depleted *Chlorella* cells is also unclear.

To address this question, we examined the time course of total phosphate (total-P) and poly-P accumulation under S- and P-depleted conditions. Furthermore, we analyzed the ultrastructure and volumetric dynamics of S-depleted cells by transmission electron microscopy (TEM) and three-dimensional (3D)-TEM. To reveal the elemental profiles and P contents of the DB as well as non-DB regions, we performed EDX analysis using a scanning transmission electron microscope (STEM). Finally, to examine the transcriptomic responses of genes related to S metabolism, such as phosphate transporters and copper transporters/chaperones, we re-analyzed RNA-Seq data^35^ of 2-day-old cells cultured under S-depleted and -replete conditions.

## Results

### DAPI staining of poly-P in S-depleted *P. kessleri* cells

**Figure 1:**
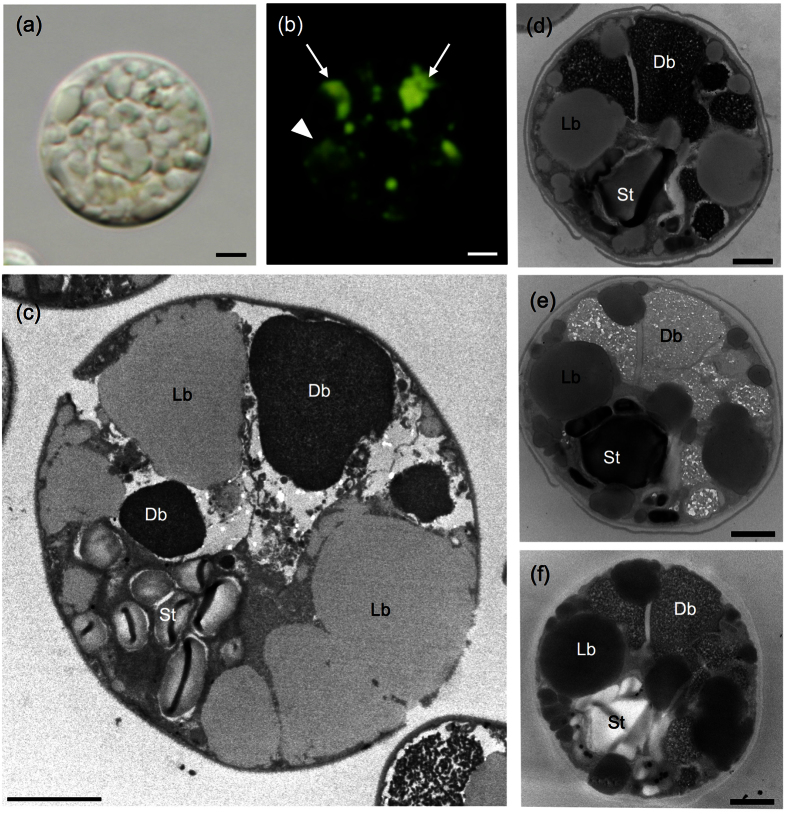
DAPI–poly-P and DBs under S-depleted conditions. Differential interference contrast image (**a**) and fluorescent image (**b**) of a DAPI-stained cell from a 6-day-old culture. (Arrows: the poly-P granules with high-fluorescence; arrowhead: blurred area of the poly-P signals) Yellow fluorescent granules indicate sites of poly-P accumulation. (**c**) General ultrastructure of a cell from a 6-day-old culture accumulating DBs and lipid bodies. (**d–f**) Triple serial sections derived from a single cell from a 5-day-old culture and subjected to the following staining methods: uranyl acetate and lead citrate staining (**d**), periodic acid-thiocarbohydrazide-silver proteinate (PAS) staining (**e**), and no staining (**f**). Db: electron-dense body, Lb: lipid body, St: starch. Bars = 2 μm (**a–c**); 1 μm (**d–f**).

DIC and fluorescent microscopy revealed that DAPI-stained poly-P granules were evident in *P. kessleri* cells cultured under S-depleted conditions or in S-deficient medium (Fig. 1a,b, Fig. S1). Numerous yellowish granules were observed using fluorescence microscopy (Fig. 1b). The poly-P granules with high-fluorescence (Fig. 1, arrows) resembled the distribution of the DBs in terms of their shape and position (Fig. 1c); however, some multiple poorly defined ‘blurred’ areas were also observed (Fig. 1, arrowhead), of which identical structure was not clear in TEM. Under S-depleted conditions, DBs accumulated concomitantly with lipid bodies (Fig. 1c). The DBs were conspicuous, and varied in size and position. Large DBs were frequently located at the cell periphery (Fig. 1c).

To assess the chemical composition of the DBs, we used the following three staining methods; uranyl acetate-lead citrate^36^, periodic acid-thiocarbohydrazide silver proteinate (PAS), and no staining (Fig. 1d–f). PAS stains starch and sugars^37^. The electron density of triple serial sections derived from the same cell can be compared using these staining methods. Following staining with uranyl acetate and lead citrate, the DBs were highly electron-opaque (Fig. 1d), whereas they were negatively stained with PAS (Fig. 1e). A non-stained section exhibited relatively electron-opaque DBs (Fig. 1f), indicating that the DBs have affinity for osmium tetroxide (OsO_4_). As expected, starch grains were strongly PAS-stained (Fig. 1e), and lipid bodies had high affinity for OsO_4_ (Fig. 1f). Therefore, the DBs contained organic compounds other than sugars and lipids.

### Lipid and P accumulation are accelerated under S-depleted conditions

**Figure 2:**
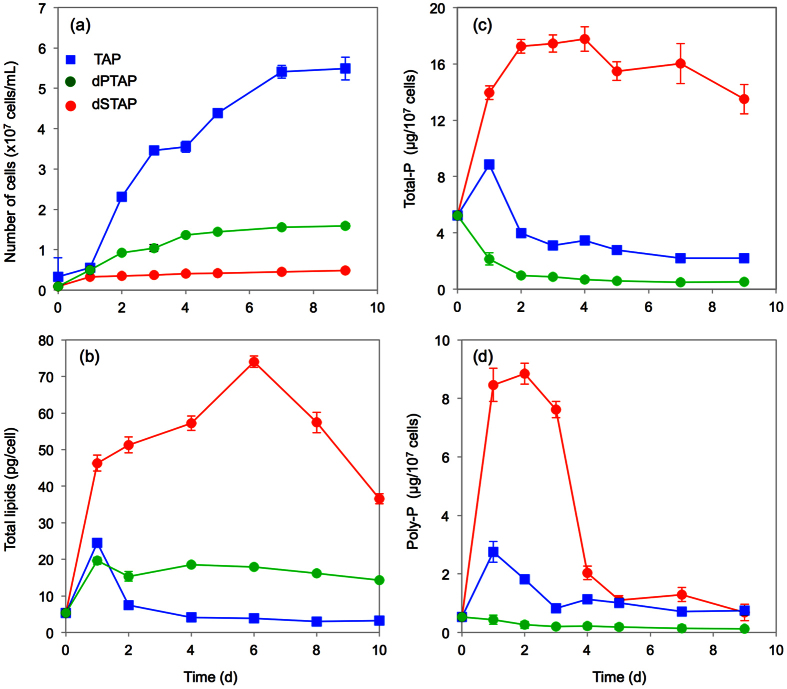
Lipid and P accumulation is accelerated by S deficiency. (**a**) Growth curves of batch cultures of *P. kessleri* in TAP (blue square), dPTAP (green circle), and dSTAP (red circle) medium. (**b**) Total lipid accumulation in batch cultures in TAP, dPTAP, and dSTAP medium. (**c**) Total phosphate (total-P) accumulation in batch cultures in TAP, dPTAP, and dSTAP medium. (**d**) Poly-P accumulation in batch cultures in TAP, dPTAP, and dSTAP medium. Values are means ± standard deviation (S.D.) of four independent assays from the same batch culture.

To eliminate any influence of the light–dark cycle on Pi accumulation, cultures were irradiated with continuous light in the present assay. In nutrient-replete Tris-acetate-phosphate (TAP) medium, exponential growth was observed in 1-day-old batch cultures, and the culture reached stationary phase after 7 d (Fig. 2a). In the S-depleted (dSTAP) and P-depleted (dPTAP) cultures, growth was repressed compared to that in TAP culture (Fig. 2a), but growth in dSTAP was compromised to a greater degree (Fig. 2a). No clear stationary phase was evident in the dSTAP culture: the number of cells increased gradually beginning at day 1 through the end of the culture period (Fig. S2). Hereinafter, 0–2 d, 3–6 d, and >7 d of culture are referred to as the early, middle, and late stages of culture, respectively.

We monitored the time-course of total lipid content during batch culture (Fig. 2b). Under S-depleted conditions, the lipid content increased from the initial day and peaked at 6 d (Fig. 2b). Thereafter, the lipid level in the dSTAP culture decreased gradually. In the dPTAP culture, total lipid levels were slightly higher than those under TAP culture in the middle and late stages (Fig. 2b).

To elucidate the P dynamics during batch culture, we determined incorporated total-P levels using a molybdenum-blue assay (Fig. 2c). After the transfer of cells to dSTAP medium, the total-P amount increased rapidly from day 0, plateaued at 2 d, and then decreased slightly after 4 d. In the TAP culture, total-P levels peaked at 1 d, then decreased from 1 to 2 d, and remained stable thereafter. At the end of the batch (9-day-old culture), 79.9% and 95.5% of phosphate still remained in media of the TAP and dSTAP, respectively (Fig. S3), indicating that phosphate is not a limiting factor for the growth. In dPTAP culture, the total-P level decreased from 0 to 2 d, and was absent thereafter (Fig. 2c).

We also examined the time-course of poly-P levels (Fig. 2d). In the dSTAP culture, poly-P accumulated rapidly from 0 to 1 d, and a high concentration was maintained from 1 to 3 d. Total-P accumulation was fivefold greater than that in TAP culture at 2 d, and almost half of the Pi had accumulated as incorporated poly-P at this time point. After 3 d, the amount of poly-P decreased rapidly, and did not change after 4 d. Although a slight peak in poly-P levels in the TAP culture at day 1 was observed, no differences were observed between the TAP and the dPTAP cultures in the middle and late stages.

### 3D-TEM and volumetric dynamics of DB and lipid bodies

To investigate the ultrastructure and volumetric dynamics of the cells in the middle stage of culture, we conducted a serial-section-based 3D-TEM analysis. We compared 3D-structures of zero-control, starch cells (starch-containing cells), and lipid cells (lipid-containing cells) in the middle stage of culture. The zero-control cell represents a 4-day-old culture under nutrient-replete conditions (stress-free conditions), which is same culture age as the pre-culture. Starch and lipid cells correspond 6-day-old cell grown in S-deficient medium (dSTAP) under a 12:12 h light:dark photoperiod (starch cell), or under continuous light (LL) (lipid cell).

**Figure 3:**
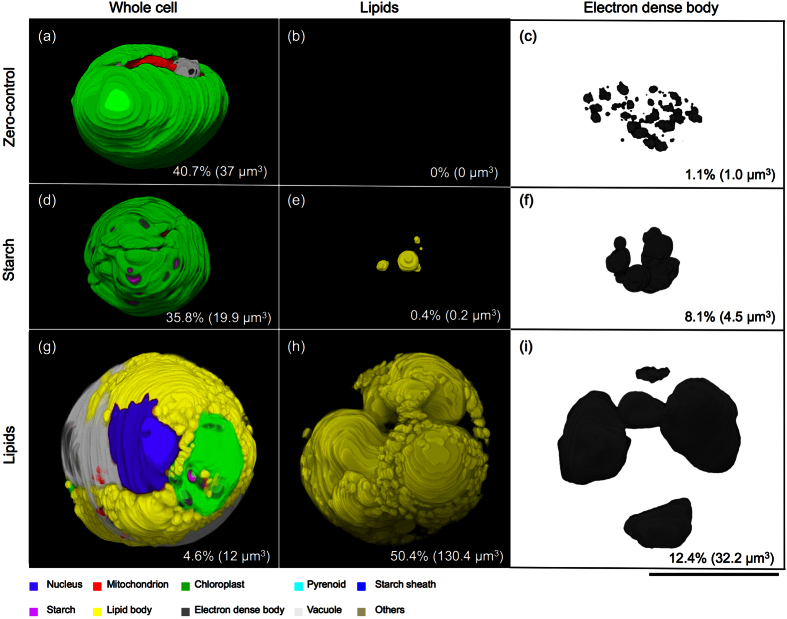
Lipid hyper-accumulation induces the accumulation of DBs. (**a–c**) 3D-TEM images of a zero-control cell from a middle stage culture in TAP medium. (**d–f**) 3D-TEM images of a starch cell cultured under S-deficient conditions in a 12:12 h light:dark photoperiod (LD). (**g–i**) 3D-TEM images of a lipid cell cultured under S-deficient conditions in continuous light (LL). Numbers represent means of volumes (n = 2) relative to that of the whole cell (%); parentheses enclose the absolute volumes (μm^3^) of chloroplasts (**a,d,g**), lipids (**b,e,h**) and DBs (**c,f,i**). All subcellular components are denoted as indicated in the legend. See also Movies S1–S10. Scale bar = 5 μm.

In the zero-control cell, a cup-shaped chloroplast was evident, and neither lipid bodies nor starch granules were observed (Fig. 3a,b; see also Movies S1,2–S3). The DBs were small granules localized in vacuoles (Fig. 3c). Based on the 3D-TEM volumetric analysis, the volume of the chloroplast and DBs of the zero-control cell contributed 40.7 and 1.1%, respectively, to the total cell volume.

In the starch cell, many starch grains and few lipid bodies were observed (Fig. 3d,e; see also Movies S4,5–S6). In this cell, the DBs were larger than those in the zero-control cell (Fig. 3f). Based on the volumetric analysis, the volumes of the chloroplast, DBs and lipids contributed 35.8, 8.1, and 0.4%, respectively, to the total cell volume.

The lipid cell exhibited a highly conspicuous degenerated chloroplast at the cell periphery, and concomitant hyper-accumulation of lipids was observed (Fig. 3g,h; see also Movies S7,8–S10). At this stage, fewer DBs were present but were well developed and conspicuous in the vacuoles (Fig. 3i). Based on the volumetric analysis, the volumes of the chloroplast, DBs, and lipids contributed 4.6, 12.4, and 50.4%, respectively, to the total cell volume. The 3D-TEM data indicate that lipid hyper-accumulation co-occurs with DB accumulation and that the continuous-light conditions enhanced the effect of sulfur deficiency stress.

### P is present in DBs under S-depleted conditions

**Figure 4:**
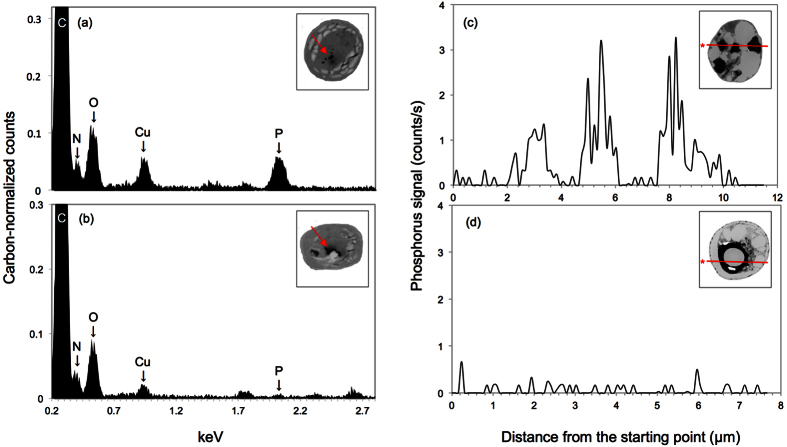
DBs in *P*. *kessleri* cells contain P in dSTAP medium, but not dPTAP medium. EDX spectrometry of the DBs of cells cultured in dSTAP (**a**) and dPTAP (**b**) medium. Vertical axis denotes carbon-normalized relative counts and the horizontal axis denotes energy as kilo-electronvolts (keV) (**a,b**). Intracellular P accumulation in dSTAP medium (**c**) and dPTAP medium (**d**) was analyzed linearly by EDX. (Insets) STEM images showing the sites (red arrows or lines) of dot analysis in a and b or line analysis in c and d. Asterisks in c and d show the starting point of the EDX linear analysis. See the supplementary data for the EDX analysis of cells cultured in TAP medium and the background.

To determine the elemental profiles of the DBs, we performed EDX analysis using STEM (Fig. 4). Carbon (C) and oxygen (O) were detected as major peaks under all conditions as well as in the background (Fig. 4a,b; Fig. S4). In the dSTAP cultures, a P peak was clearly detected (Fig. 4a); however, a low P peak was detected in the DBs of cells cultured in dPTAP or in the background (Fig. 4c, Fig. S4). A nitrogen (N) peak was detected in cells cultured in the TAP, dSTAP and dPTAP media (Fig. 4a,b; Fig. S4). A copper (Cu) peak was detected in cells cultured in dSTAP (Fig. 4b), which might be contamination from TEM operation. It remains unknown whether there was a larger difference in oxygen amount between +/−S cells, even if Pi was present.

To assess the presence of elemental P in the DB and non-DB regions of the same cell, an EDX analysis was performed linearly across the section (Fig. 4c,d). Under S-depleted conditions, P was detected in the DB, but not in other non-DB regions of the cytoplasm (Fig. 4c). No P was detected in cells cultured in dPTAP, even in the DB region (Fig. 4d). Considering that poly-P was detected under S-depleted conditions as well as TAP culture (Fig. 2d, Fig. S4), these results indicate that P is present only in the DBs as phosphate; therefore, the DB may be a site of poly-P accumulation. In addition, the DBs maintained their electron density even in the absence of P, indicating that P-free-DBs could be present under phosphorus-depleted conditions.

### Ultrastructural changes of DBs under S-depleted conditions

**Figure 5:**
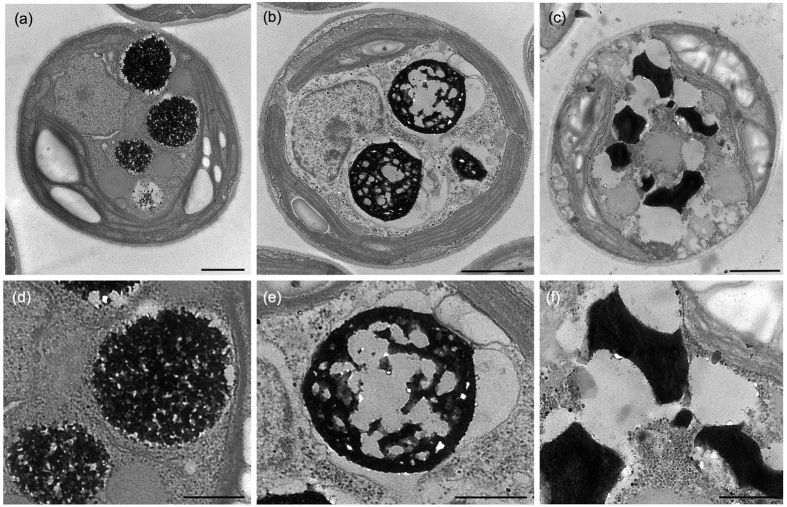
Effect of culture age on DB ultrastructure. General ultrastructure of cells from 2-day-old (**a**), 6-day-old (**b**), and 12-day-old (**c**) S-deficient culture. Higher magnification images of DBs in cells from 2-day-old culture (**d**), 6-day-old culture (**e**) and 12-day-old culture (**f**). Bars = 1 μm (**a–c**); 500 nm (**d–f**).

P-free-DBs were observed under P-depleted conditions, as mentioned above. Because the poly-P level decreased markedly after culture for 4 d in S-deficient medium (Fig. 2d), we investigated ultrastructural changes during this period. We focused on the ultrastructure of *P. kessleri* cells in the early (2 d), middle (6 d), and late (12 d) periods of culture under S-deficient conditions (Fig. 5). In the early stage of culture, the vacuoles were filled with electron-dense material, within which electron-lucent dot-like structures were observed (Fig. 5a,d). In the middle stage of culture, the electron-lucent region in the DBs had expanded and was frequently located in the center of the DBs (Fig. 5e). In the late stage of culture, the DBs were compressed in the vacuoles (Fig. 5c,f); no dot-like structures were evident. These data suggest ultrastructural variation in the DBs, which may be associated with the presence or absence (hydrolysis) of poly-P.

### RNA-Seq analysis of cells grown in S-deficient culture

**Figure 6:**
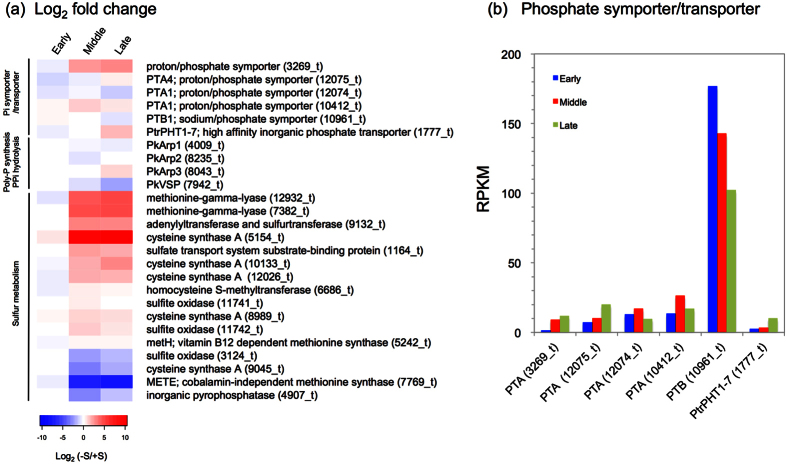
Transcriptome response to S deficiency by RNA-seq analysis. (**a**) Heat map showing the log_2_-scaled fold-changes of RPKM of selected genes (S metabolism, Pi symporter/transporters, poly-P synthesis/PPi hydrolysis) in 2-day-old cultures under S-deprived conditions relative to those under S-replete conditions at the early, middle, and late stages of culture. (**b**) Absolute values of RPKM for genes related to phosphate symporter/transporters. PTA: proton/phosphate symporter, PTB: sodium/phosphate symporter, PtrPHT1-7: high-affinity inorganic phosphate transporter.

To assess the transcriptomic response to the presence and absence of S, we reanalyzed the *Parachlorella* RNA-Seq dataset^35^ at the early, middle, and late stages of culture. A heat map showing the log_2_-fold changes of reads per kilobase of exons per million mapped sequence reads (RPKM) values in the early, middle, and late cultures under S-deficient conditions compared to those in the 2-day-old TAP culture is shown in Fig. 6a.

Among the six representative genes of the phosphate symporter/transporter family (the sodium/phosphate symporter is a Na^+^ -Pi cotransporter, and the proton/phosphate symporter is an H^+^ -Pi cotransporter), three were upregulated markedly during the middle and late stages of culture under S-deficient conditions (Fig. 6a). We also compared the absolute RPKM values (Fig. 6b). Among the phosphate symporter/transporter gene family members, *PTB* (10961_t; accession no. LC093963) showed RPKM values that were markedly higher than those of the other genes, suggesting that *PTB* is a key phosphate transporter gene in *P. kessleri*. Based on the BLASTP search against the *Parachlorella* genome^35^, *PTB* (10961_t) is a single copy gene in the genome. It should also be noted that *PTB* transcript levels decreased during the culture period (Fig. 6B), which was consistent with the P-accumulation in the early stage of culture under S-depleted conditions. We observed few changes in the *PkArp* genes that are related to polyphosphate synthesis (except for *PkArp3* at the late stage; Fig. 6a). We also found a putative poly-P hydrolysis gene (PkVSP; 7942_t) in the *Parachlorella* genome. Although *PkVSP* was slightly downregulated at the late stage, we observed few changes in transcript levels at the early and middle stages (Fig. 6a).

We also focused on genes related to polyphosphate synthesis/degradation and sulfur metabolism, as the present experiment was performed under S-depleted conditions. Of the 15 genes implicated in starch, cysteine, and methionine metabolism, the majority were induced in response to S deficiency at the middle and late stages of culture (Fig. 6b). Although one copy of *cysteine synthase A* (9045_t) was downregulated, the other four *cysteine synthase A* genes were highly induced in response to sulfur starvation. The *cysteine synthase A* (5154_t) gene was upregulated 1425-fold (−S/+S) (Fig. 6a).

## Discussion

We determined the site of poly-P accumulation in *Parachlorella kessleri* cells under stress conditions. First, we compared the distribution of the DBs and poly-P accumulation sites of cells cultured under S-depleted conditions using fluorescence and transmission microscopy. The subcellular distributional pattern of the DAPI–poly-P complex granules with higher fluorescence was similar to that of the DBs; large granules were localized to the cell periphery while small granules were scattered irregularly (Fig. 1b,c). In addition, serial section analysis showed that DBs had affinity for heavy metals but were negative for PAS staining, indicating that DBs contain organic compounds other than sugars and starch. Thus, we infer that the DBs are the sites of poly-P accumulation.

Moreover, we determined the subcellular P accumulation by EDX analysis (Fig. 4, Fig. S4). P was found in the DBs under S-deficient conditions as well as in TAP medium, whereas no P was found in the DBs under P-deficient conditions. The acidocalcisome, a term commonly used to describe an organelle in *C. reinhardtii,* trypanosomatids, and related protists such as *Toxoplasma gondii*^38^, also contains DBs. This organelle contains P in the form of Pi and poly-P as well as calcium (Ca) and other elements such as iron (Fe)^39^,^40^,^41^. In this study, Ca (Kα = 3.6905 keV, Lα = 0.3413 keV) and Fe (Kα = 6.3996 keV, Lα = 0.7048 keV), major metal elements of acidocalcisomes, were not detected under any of the culture conditions tested (see Fig. S5), indicating that the DBs detected in *P. kessleri* may be different from acidocalcisomes.

We also examined P accumulation in the non-DB region by linear EDX analysis of cell sections (Fig. 5). P was found only in the DBs in cells cultured in dSTAP medium, and no signal was detected in the DB or non-DB regions in cells cultured under phosphorus-deficient conditions. Based on the EDX analysis and Pi assay data (Fig. 2d), we infer that P is incorporated rapidly into S-depleted cells, and accumulated as poly-P into the DBs, indicating that DBs are sites which serve as Pi reservoirs in *P. kessleri*. However, the nature of the interaction of poly-P with electron dense materials remains to be elucidated. Based on the 3D-TEM data, lipid accumulation may be correlated with increasing DB volume. Pi and lipid accumulation may be a response to S-deficiency stress, the effect of which was enhanced by continuous light. However, the metabolic connection between poly-P and lipid accumulation remains unclear.

Growth of *P. kessleri* was inhibited markedly in dSTAP. Marked lipid accumulation occurs concurrently under conditions of S deficiency. This is consistent with previous reports that lipid levels in *Parachlorella* and *Chlorella* species increased under conditions of S deficiency^32^,^33^. P-depleted medium also inhibited cell growth, but to a lesser degree than S deficiency (Fig. 2). Our data also suggest that P-deficient cells accumulate lower total lipid levels.

Total-P accumulation increased markedly during the early stage of culture under S-depleted conditions (Fig. 2). With regard to the ratio of the amounts of poly-P to total-P, 43% of incorporated Pi was transformed into poly-P as a storage substance by the end of the early stage (3-day-old culture) (Fig. S3). After culture for 4 d, the poly-P level decreased markedly; however, the total-P level remained roughly constant (Fig. 2). This discrepancy between the poly-P and total-P levels may be explained by hydrolysis of long-chain poly-P in the DB into Pi during the middle and later stages (≥4 d) of culture.

Poly-P functions as a protein-protective chaperone for adaptation to stress^42^,^43^. In this study, total-P and poly-P accumulation accelerated during the early stage of culture under S-deficient conditions. Mutants of *Escherichia coli* that fail to synthesize polyphosphate kinase (PPK) and lack long-chain poly-P fail to survive in the stationary phase and lose their resistance to stresses^44^,^45^. In *Trypanosoma cruzi*, the concentration of poly-P changes drastically during growth and differentiation. In addition, a response to stress was reported in the halotolerant alga *Dunaliella salina*^46^. Therefore, Pi and poly-P accumulation in *P. kessleri* under S-deficient conditions may also be due to the stress response under S-deficient conditions. Based on the ultrastructural findings and physiological data, we infer that poly-P accumulates in DBs, which function as reservoirs during the early stage of culture.

To examine the transcriptomic response of genes of interest, we reanalyzed the *Parachlorella* RNA-Seq dataset^35^ at the early, middle, and late stages of culture under S-deprived and -replete conditions. Because the present experiment was performed under S-depleted conditions, we also focused on genes related to sulfur metabolism. The vast majority of genes associated with Pi and S metabolism were upregulated in the middle and late stages of culture (Fig. 6). Notably, *PAT1* (*proton/phosphate symporter 1*, 3269_t), *PAT4*, and *cysteine synthase A* (5154_t) were highly upregulated in the middle and later stages. The present data on S acquisition and assimilation to cysteine are consistent with a previous study in *Chlamydomonas*^47^.

We could not find *PPK* (polyphosphate kinase) and *PPX* (exopolyphosphatase) homologs in the *Parachlorella* genome^35^. We searched for other candidates of poly-P synthesis/degrading genes in the genome and found a homolog of *Arp* (a gene for actin-related protein): *PkArp1* (4009_t), *PkArp2* (8235_t), and *PkArp3* (8043_t). The Arp complex is an enzyme that can polymerize an actin-like filament concurrent with its synthesis of a poly-P chain^48^. We also found a homolog of *VSP* (a vacuolar soluble pyrophosphatase gene), which is a putative gene for poly-P and pyrophosphate hydrolysis^49^. We observed few changes in expression levels of *PkArps* and *PkVSP* under the S-deprivation condition, except in the late stage (Fig. 6a). This result may suggest that poly-P accumulation under S-deprivation is more affected by luxury Pi uptake than by poly-P synthesis/degradation. Further physiological experiments are needed to confirm this conclusion.

**Figure 7:**
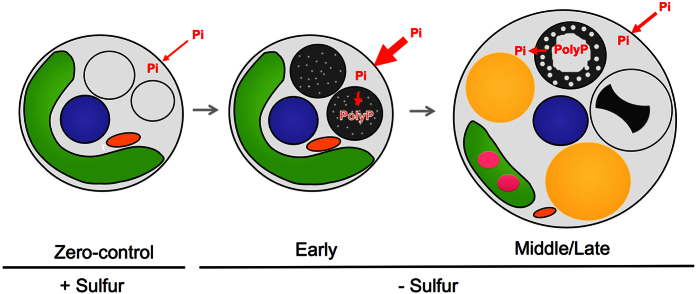
Schematic illustration of proposed dynamics of Pi and DB ultrastructure in S-deficient culture. In the S-replete condition, few dense bodies are observed in vacuoles. In the early stage of culture under S deprivation, transportation and accumulation of phosphate (Pi) is accelerated and incorporated into DBs as poly-P. At this stage, the DBs contain electron-opaque materials with small dot-like electron-lucent structures. In the middle/late stage under S deprivation, the electron-lucent regions are expanded, the density of the DBs decreases, and poly-P undergoes partial hydrolysis to Pi. In the late stage, the DBs are highly compressed and Pi may be released to the cytosol and so be absent from the DBs. Arrows indicate the flow of Pi. The thickness of the arrows represents the transcriptomic data. Green, chloroplast; purple, nucleus; magenta, starch; yellow, lipid bodies, gray/black, DB; orange, mitochondria.

Although the ultrastructure of the DBs in *C. pyrenoidosa* has been reported^22^, the ultrastructural changes of the DBs in *Chlorella* species over time remained largely unknown. Based on our findings, we propose the following model of dynamics of poly-P and ultrastructural changes of the DB under S-deficient conditions (Fig. 7). In the early stage of culture, poly-P is accumulated rapidly in the DBs. This is supported by the RNA-Seq data; transcript levels of *PTB*, which encodes a key Pi transporter, decreased during the culture period (Fig. 6b).

The electron-lucent regions in the DBs expanded in cells at the middle stage of culture. In cells during the late stage of culture, the DBs were compressed and localized to the vacuole (Fig. 7). The compressed DBs were electron-opaque and lacked the dot-like structures present in the early and middle stages. Intriguingly, the ultrastructure of the compressed DBs was similar to that of the DBs in cells cultured under P-deficient conditions (Fig. S6), suggesting them to be of the same type as the Pi-free-DBs present in cells in the later stage of culture under S-deficient conditions. Taken together, our findings show that DBs in *P. kessleri* are sites of accumulation of poly-P and that DB ultrastructure varies considerably according to the presence or absence of poly-P.

In this study, our data indicate that P accumulation increased markedly under sulfur-depleted conditions, and that DBs are a polyphosphate accumulation site in the green alga *Parachlorella kessleri*. In addition, considerable ultrastructural variation was observed in DBs according to the amount of P. The TEM observations revealed marked ultrastructural variations in the DBs with or without poly-P, as well as hyper-accumulation of lipids. Based on our findings, we propose that the DBs are a site of poly-P accumulation during the early stage of culture under S-depleted conditions. Because *P. kessleri* achieves high poly-P content, as well as high biomass and lipid production, this species has potential as a phosphate accumulating organism.

## Methods

### Strains and growth media

*Parachlorella kessleri* strain NIES-2152 was obtained from the Microbial Culture Collection, National Institute for Environmental Studies, Tsukuba, Japan (http://mcc.nies.go.jp/). Precultures were grown for 4 d in a test tube (TEST 30 NP; IWAKI, Tokyo, Japan) in 30 mL of Tris-acetate-phosphate (TAP) medium (Table S1) at 23 °C under a 12 h light (L):12 h dark (D) cycle. The culture was incubated under a photon flux of ~100 μmol m^−2^ s^−1^ with agitation using a magnetic stirrer (MGM-66, Shibata, Tokyo, Japan) at approximately 100 rpm. For nutrient limitation, sulfur (SO_4_^2−^)-deficient TAP (dSTAP; Table S2) and phosphorus-deficient TAP (dPTAP; Table S3) media were used.

### Transmission electron microscopy

Five-day-old cultures grown in dSTAP (LL) were fixed with 2.5% glutaraldehyde (GA) for 2 h at room temperature (r.t.). To permeate GA, the cell suspension was microwaved for 20 s on ice at the beginning of the fixation. The cells were washed four times with 0.1 M sodium cacodylate buffer (pH 7.2). After centrifugation and removal of the supernatant, the pellet was post-fixed with 1% osmium tetroxide for 2 h at r.t. Dehydration, embedding, and uranyl acetate-lead citrate staining methods followed the procedures described by Wayama *et al.*^50^. For silver-proteinate staining, sections were treated with periodic acid-thiocarbohydrazide and then stained with silver proteinate^37^.

### Energy dispersive X-ray spectroscopy

Six-day-old cultures grown under LD conditions were fixed with 2.5% glutaraldehyde. Dehydration and embedding methods followed the procedures described by Wayama *et al.*^50^. Ultrathin sections were cut on a Reichert Ultracut S ultramicrotome (Leica, Vienna, Austria) using a diamond knife, mounted on a copper grid and observed without staining using an HT7700 electron microscope (Hitachi, Tokyo, Japan) operating at 100 kV. The elemental composition of DBs was determined by energy-dispersive X-ray spectroscopy (EDS; X-Max80, Horiba, Kyoto, Japan). The measurement points or lines are indicated in the dark-field STEM images.

### Poly-P detection by staining with 4′,6-diamidino-2-phenylindole (DAPI)

Poly-P can be stained by high concentrations of DAPI. The peak emission wavelength of the resulting DAPI-poly-P complex is ~525 nm; *i*.*e*., a yellow or greenish-yellow color. For poly-P observations, cells were fixed with 1% GA for 5 min at r.t. and washed with phosphate-buffered saline (pH 7.4) containing 0.1% Triton X-100. Cell suspensions were stained with DAPI at a final concentration of 100 μg/mL. After incubation for 1 h at r.t., cells were observed using a BX-60 fluorescence microscope (Olympus, Tokyo, Japan) equipped with a DP70 CCD camera (Olympus, Tokyo, Japan). For poly-P detection, UV excitation was used and emission at >420 nm was detected using a U-MWU filter cube.

### Growth, lipids, and total phosphate (total-P) and poly-P assays

Cell numbers were determined using a particle counter (CDA-1000, SYSMEX, Kobe, Japan). The lipid assay described by Takeshita *et al.* was used^32^. For a total-P assay, 10 mL of culture were sampled, centrifuged at 2,500 × *g* for 5 min, and the pelleted cells were collected. The pellet was resuspended in distilled water (DW) and washed by centrifugation at 2,500 × *g* for 5 min. The pellet was resuspended in 1 mL of DW, and the cells were disrupted by vortexing with glass beads for 15 min at 4 °C. For hydrolysis to orthophosphate, 200 μL of 4% (w/v) potassium persulfate were added to the sample, which was then autoclaved at 121 °C for 30 min. The lysate was subjected to a molybdenum blue assay (see below).

For a poly-P assay, 10 mL of culture were sampled, centrifuged at 2,500 × *g* for 5 min, and the pelleted cells were collected. The pellet was resuspended in 1 mL of 5% sodium hypochlorite, and the cells were disrupted by vortexing with glass beads for 15 min at 4 °C. After centrifugation at 14,000 × *g* for 3 min, the supernatant was removed, and then the pellet was resuspended in 1 mL of 5% sodium hypochlorite, centrifuged at 14,000 × *g* for 3 min, and the supernatant was again removed. Next, the pellet was resuspended in 100 μL of DW, followed by incubation for 5 min at r.t. and centrifugation at 14,000 × *g* for 3 min; the supernatant was then decanted. Again, the pellet was resuspended in 100 μL of DW, followed by incubation for 5 min at r.t. and centrifugation at 14,000 × *g* for 3 min; the supernatant was then collected. The retrieved supernatant (200 μL) containing poly-P was precipitated with 90% ethanol (final conc.), centrifuged at 14,000 × *g* for 10 min, and the supernatant removed. The poly-P pellet was then dissolved in 50 μL of DW. For hydrolysis to orthophosphate, 100 μL of 4% (w/v) potassium persulfate were added to the poly-P solution, followed by autoclaving at 121 °C for 30 min and subjection to a molybdenum blue assay (see below).

Orthophosphate (Pi) was assayed by the molybdenum blue method in a 96-well microplate (IWAKI, Tokyo, Japan). A diluted phosphate sample (200 μL) was pipetted into the wells, and 8 μL of ammonium molybdate tetrahydrate solution [1.2% (w/v) with 4.8 mg potassium antimonyl tartrate sesquihydrate and 16 mL sulfuric acid per 100 mL DW] and 2 μL of 7.2% (w/v) L-ascorbic acid solution were added, and the solution mixed. After incubation for 20 min at r.t. in the dark, absorbance at 880 nm was read using a microplate reader (Viento nano; BioTek Japan, Tokyo, Japan). Phosphate Ion Standard Solution (Wako Pure Chemical Industries, Osaka, Japan) was used for the standard curve, the linearity range of which was 0 to 2 mg NaH_2_PO_4_/L.

### Three-dimensional transmission electron microscopy (3D-TEM) analysis

For 3D-TEM analysis, we reconstructed 3D images of a 4-day-old cell grown in sulfur (S)-replete medium (TAP, no-stress condition) (zero-control cell), a 6-day-old cell grown in S-deficient medium (dSTAP) under a 12:12 h light:dark photoperiod (LD) (starch cell), and a 6-day-old cell cultured in S-deficient medium (dSTAP) under continuous light (LL) (lipid cell). The detailed procedures for TEM and 3D reconstruction are described in Wayama *et al.*^50^, with the exception of the modifications described below. Digital stacked images were displayed on a tablet device (iPad; Apple Inc., CA) and the contours of each subcellular element were traced manually in color using Procreate (ver. 2.0.2). The traced images were converted into digital images (JPG format), and 3D reconstructions were performed using the TRI/3D SRF III software (Ratoc System Engineering, Co., Ltd., Tokyo, Japan). Each subcellular volume was calculated from voxels using the TRI/3D SRF III software. Values are the means of two representative cells in each growth phase.

### RNA-Seq analysis

To focus on the transcriptomic responses of genes associated with Pi and S metabolism, we reanalyzed the *Parachlorella* RNA-Seq dataset^35^. The methods for total RNA extraction, mRNA purification, and RNA sequencing were described previously^35^. Briefly, total RNA was extracted from cultures with or without S in the early (2-day-old cells) and late (4–5-day-old cells) logarithmic and stationary phases (7–8-day-old cells). The mRNA sequencing libraries of *P. kessleri* NIES-2152 were constructed using the Ion Total RNA-Seq Kit v2 (Life Technologies, Carlsbad, CA, USA), and the libraries were sequenced using an Ion PGM Sequencer (Life Technologies). The heatmap was generated using the heatmap2 function in Gplots package v. 2.16.0 in R statistical software v. 3.1.0 (http://www.R-project.org/).

## Additional Information

**Accession Codes**: The sequencing data used in the present RNA-seq analysis have been deposited in the DDBJ database under accession numbers: LC093959 (12075_t), LC093960 (12074_t), LC093961 (3269_t), LC093962 (10412_t), LC093963 (10961_t), LC093964 (1777_t), LC093965 (12932_t), LC093966 (7382_t), LC093967 (9132_t), LC093968 (5154_t), LC093969 (1164_t), LC093970 (10133_t), LC093971 (12026_t), LC093972 (6686_t), LC093973 (11741_t), LC093974 (8989_t), LC093975 (11742_t), LC093976 (5242_t), LC093977 (3124_t), LC093978 (9045_t), LC093979 (7769_t), LC128310 (4009_t), LC128311 (8235_t), LC128312 (8043_t), LC128419 (7942_t).

**How to cite this article**: Ota, S. *et al.* Deciphering the relationship among phosphate dynamics, electron-dense body and lipid accumulation in the green alga *Parachlorella kessleri*. *Sci. Rep.* **6**, 25731; doi: 10.1038/srep25731 (2016).

## Supplementary Material

Supplementary Information

Supplementary Movie S1

Supplementary Movie S2

Supplementary Movie S3

Supplementary Movie S4

Supplementary Movie S5

Supplementary Movie S6

Supplementary Movie S7

Supplementary Movie S8

Supplementary Movie S9
