# Supplementary material for: Deciphering the relationship among phosphate dynamics, electron-dense body and lipid accumulation in the green alga Parachlorella kessleri
Source: Sci Rep. 2016 May 16;6:25731. doi: 10.1038/srep25731 (PMC4867602; doi:10.1038/srep25731)
Supplement: Supplementary Information [file srep25731-s1.pdf]

Supplementary information

Deciphering the relationship among phosphate dynamics, electron-dense body and lipid accumulation in the green alga *Parachlorella kessleri*

Shuhei Ota[1,2], Mai Yoshihara[1], Tomokazu Yamazaki[1,2], Tsuyoshi Takeshita[1], Aiko Hirata[3], Mami Konomi[4], Kenshiro Oshima[5], Masahira Hattori[5], Kateřina Bišová[6], Vilém Zachleder[6], Shigeyuki Kawano[1,2]*

[1]Department of Integrated Biosciences, Graduate School of Frontier Sciences, University of Tokyo, Kashiwa, Chiba, 277-8562, Japan. [2]CREST, Japan Science and Technology Agency, Tokyo, Japan. [3]Bioimaging Center, Graduate School of Frontier Science, University of Tokyo, Kashiwa, Chiba, 277-8562, Japan. [4]Hitachi High-Technologies Corporation, Science & Medical Systems Business Group, Nishi-shinbashi, Tokyo, 105-8717, Japan. [5]Center for Omics and Bioinformatics, Graduate School of Frontier Sciences, University of Tokyo, Kashiwa, Chiba, 277-8561, Japan. [6]Institute of Microbiology, CAS, Centre Algatech, Laboratory of Cell Cycles of Algae, Třeboň, Czech Republic.

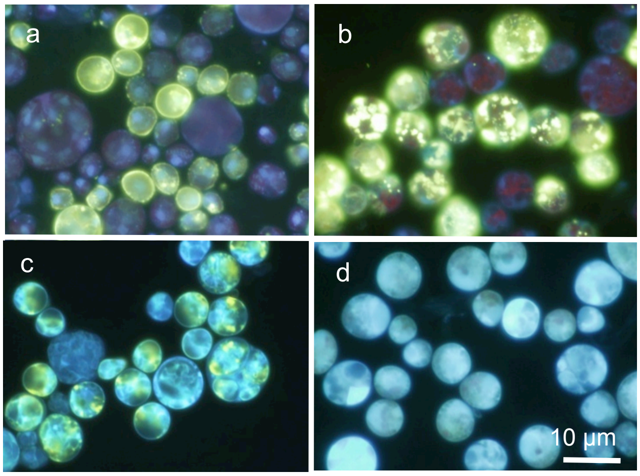

Figure S1. PolyP visualization of *P. kessleri* cells with high-concentration DAPI staining. (a) DAPI staining of cells growing on TAP medium. (b) DAPI staining of cells growing on dSTAP medium. (c) DAPI staining of cells growing on dNTAP medium (N-depleted medium). (d) DAPI staining of cells growing on dPTAP medium. Poly-P is stained yellow.

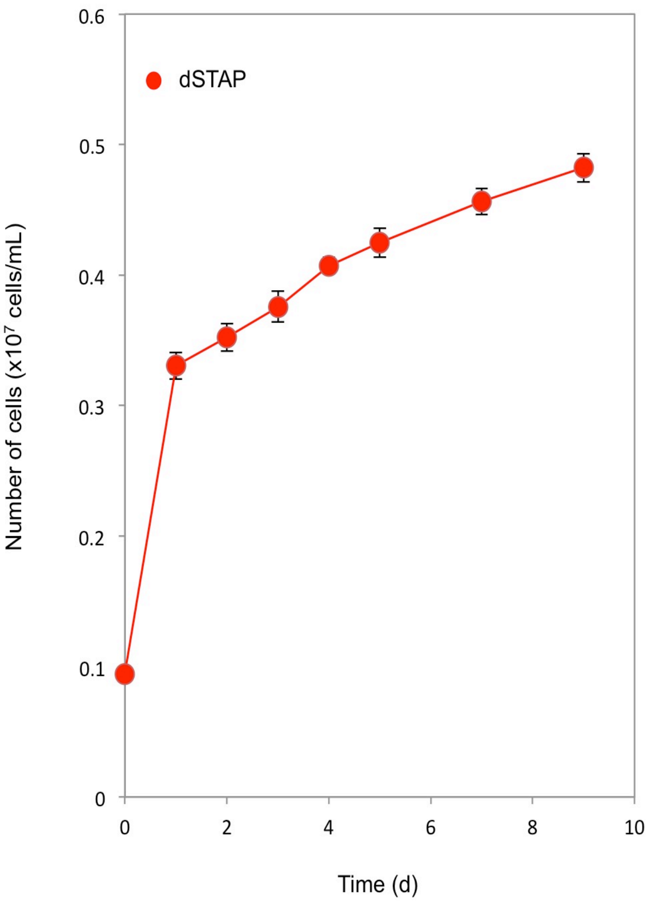

Figure S2. Rescaled representation of a growth curve in S-deficient medium. Values are means ± standard deviation (S.D.) of four independent assays from the same batch culture.

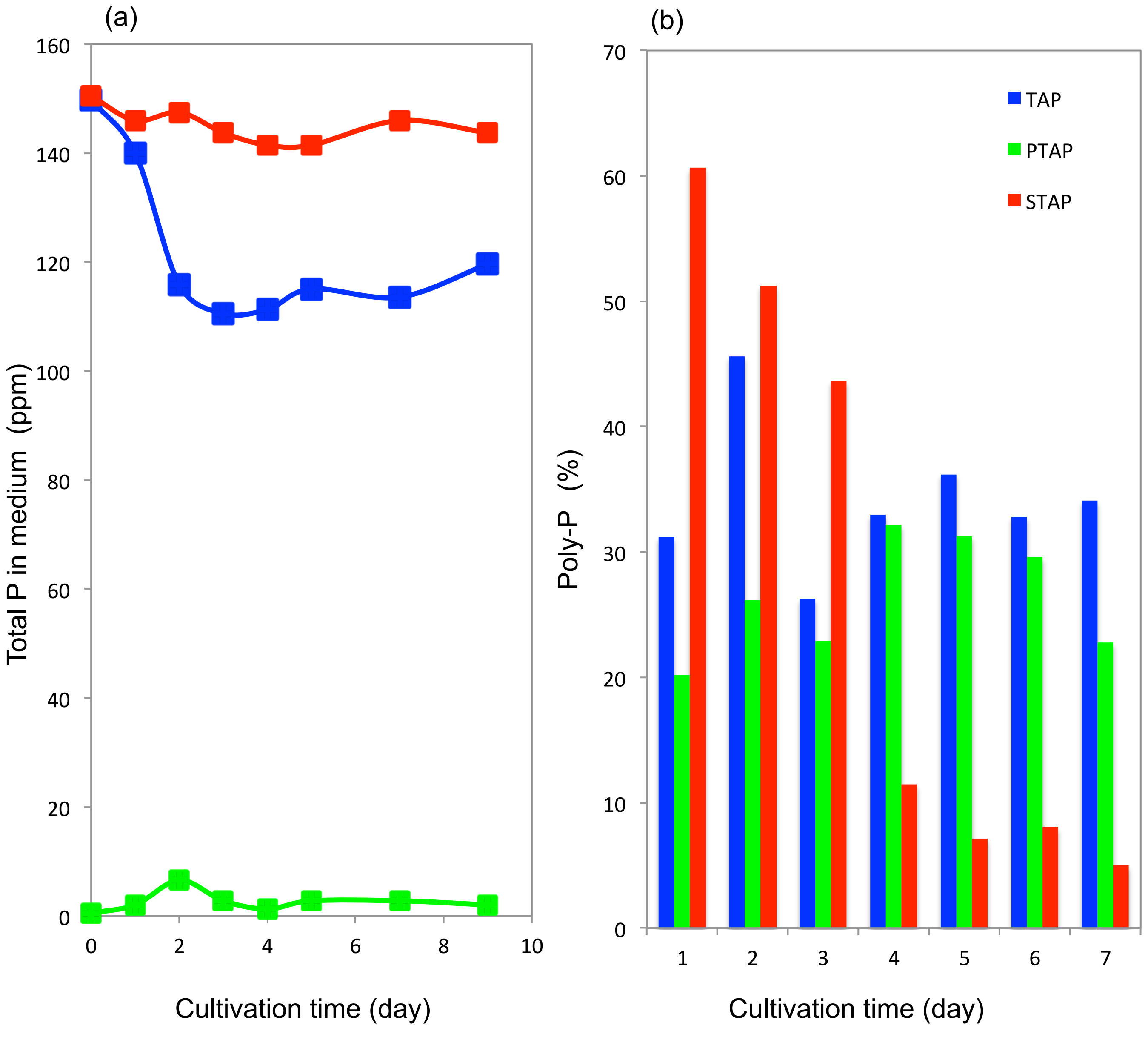

Figure S3. Remaining and stored phosphate dynamics. (a) The amount of phosphate remaining in the medium. (b) Percent of stored poly-P in each medium.

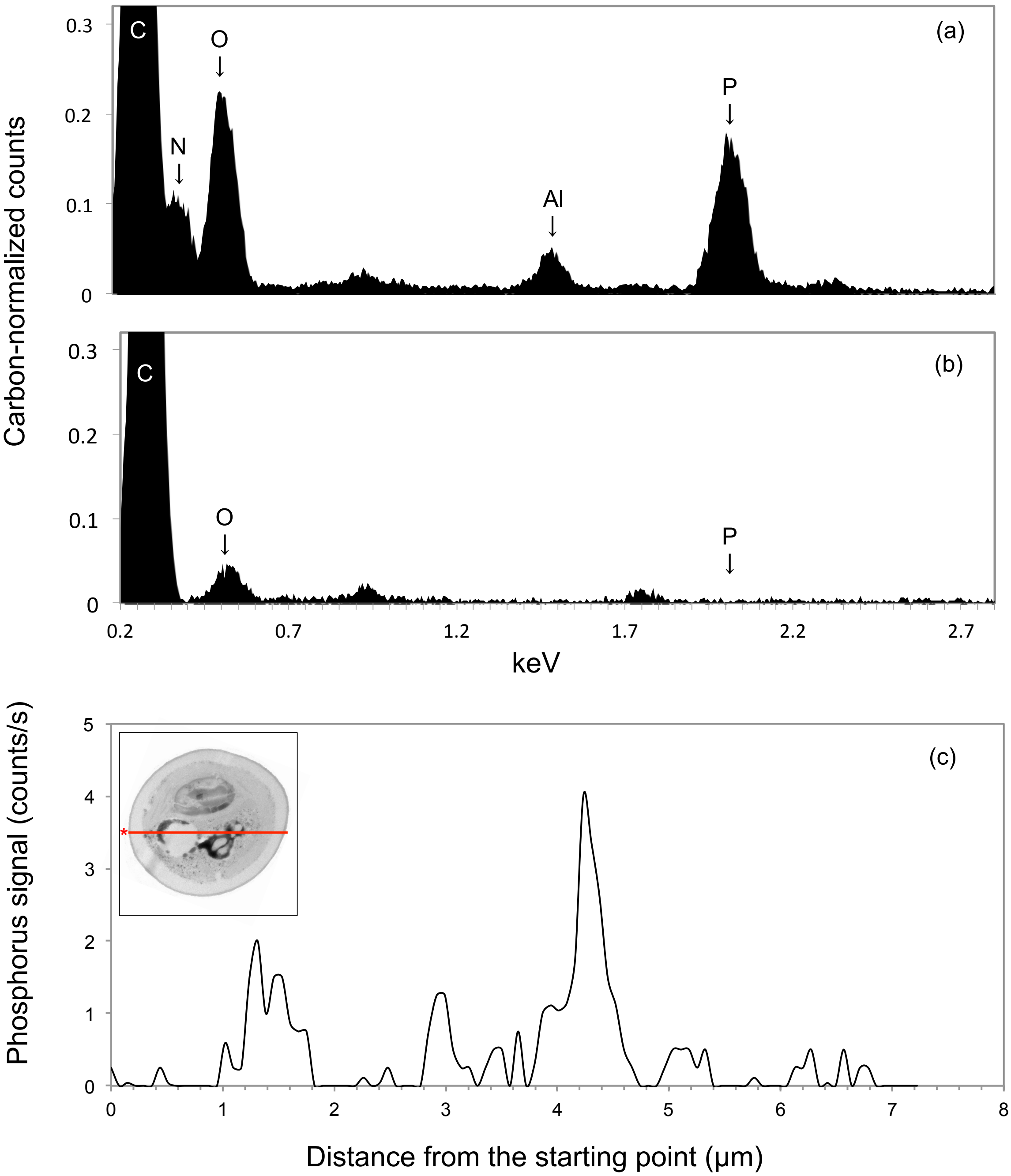

Figure S4. EDX analysis of DBs in TAP and the resin background. EDX spectrometry of DBs in cells cultured in TAP (a) and the resin background (b). P accumulation in cells cultured in TAP medium was analyzed linearly by EDX (c). (Insets) STEM images showing the site (red lines) of the line analysis in c. Asterisks in c show the starting points of the EDX linear analysis.

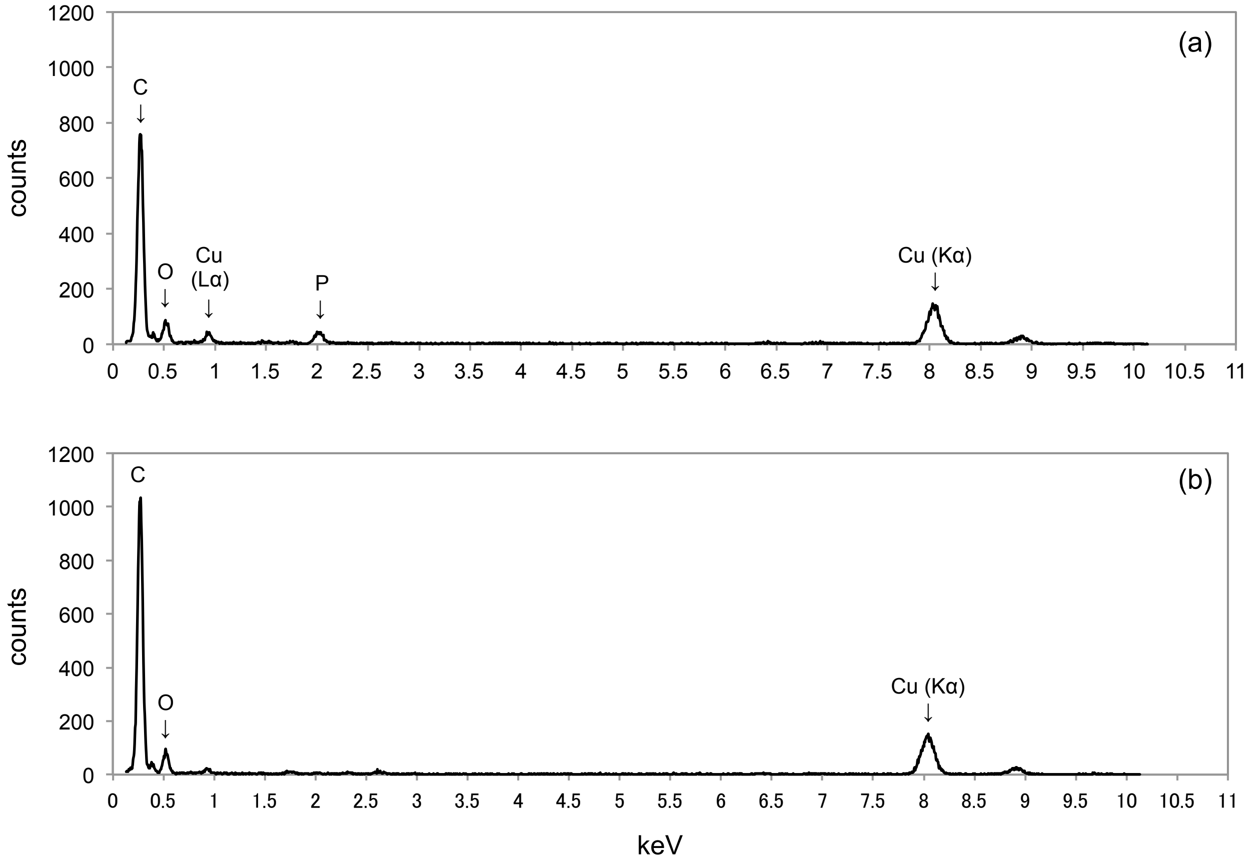

Figure S5. **Row count data from the EDX analysis.** (a) Non-normalized EDX counts corresponding to Fig. 4a (dSTAP). (b) Non-normalized EDX counts corresponding to Fig. 4b (dPTAP).

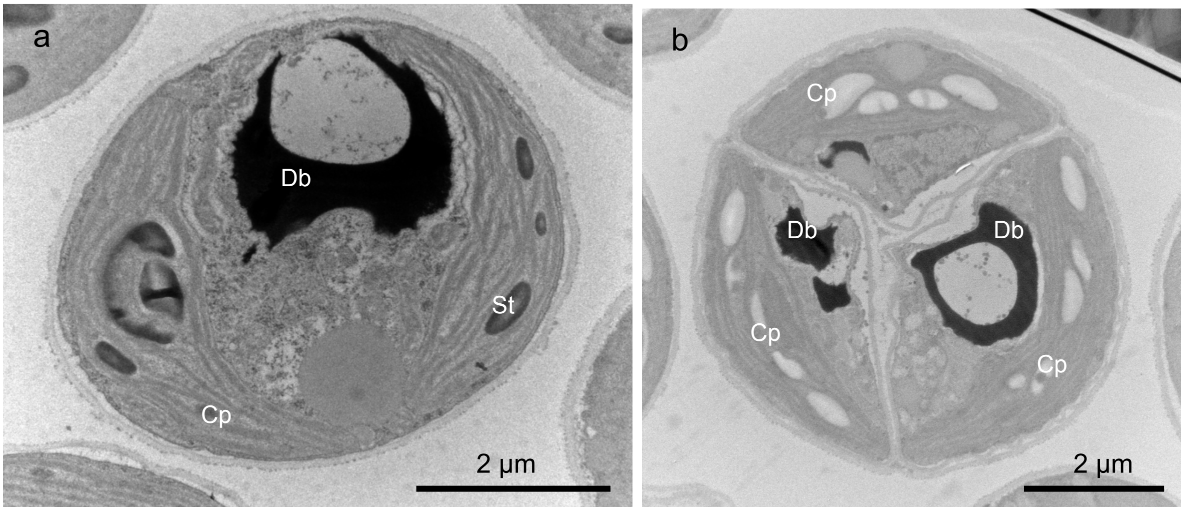

Figure S6. Ultrastructure of DBs in P-depleted cells. TEM observations of cells in 6-day-old P-depleted culture (a and b). Cp: chloroplast, Db: electron-dense body, St: starch.

Table S1. Recipe of TAP medium

Component	Stock Solution (mg/mL)	Quantity Used*
NH_4Cl	200	2.0 mL
$CaCl_2 \cdot 2H_2O$	100	0.5 mL
$MgSO_4 \cdot 7H_2O$	300	1.0 mL
K_2HPO_4	100	1.0 mL
KH_2PO_4	100	1.0 mL
Hutner's trace elements	See following recipe	1.0 mL
Acetic acid	-	1.0 mL
Tris (hydroxymethyl) aminomethane	-	2.42 g

*To prepare: make up to 1 liter with distilled water (DW). Autoclave at 121°C for 20 minutes.

Recipe of Hutner's trace elements

Component	Quantity Used (per 100 mL DW)**
$Na_2EDTA \cdot 2H_2O$	5.000 g
$ZnSO_4 \cdot 7H_2O$	2.200 g
H_3BO_3	1.140 g
$MnCl_2 \cdot 4H_2O$	0.506 g
$FeSO_4 \cdot 7H_2O$	0.499 g
$CoCl_2 \cdot 6H_2O$	0.161 g
$CuSO_4 \cdot 5H_2O$	0.157 g
$(NH_4)_6Mo_7O_{24} \cdot 4H_2O$	0.110 g
KOH	~1.6 g

**Dissolve each of the above components, and then bring the final volume up to 100 mL with DW.

Table S2. Recipe of dSTAP medium

Component	Stock Solution (mg/mL)	Quantity Used*
NH_4Cl	200	2.0 mL
$CaCl_2 \cdot 2H_2O$	100	0.5 mL
$MgCl_2 \cdot 6H_2O$	254	1.0 mL
K_2HPO_4	100	1.0 mL
KH_2PO_4	100	1.0 mL
Hutner's trace elements for dSTAP	See following recipe	1.0 mL
Acetic acid	-	1.0 mL
Tris (hydroxymethyl) aminomethane	-	2.42 g

*To prepare: make up to 1 liter with DW. Autoclave at 121°C for 20 minutes.

Recipe of Hutner's trace elements for dSTAP

Component	Quantity Used (per 100 mL DW)**
$Na_2EDTA \cdot 2H_2O$	5.000 g
$ZnCl_2$	1.040 g
H_3BO_3	1.140 g
$MnCl_2 \cdot 4H_2O$	0.506 g
$FeCl_3 \cdot 7H_2O$	0.485 g
$CoCl_2 \cdot 6H_2O$	0.161 g
$CuCl_2 \cdot 2H_2O$	0.107 g
$(NH_4)_6Mo_7O_{24} \cdot 4H_2O$	0.110 g
KOH	~1.6 g

**Dissolve each of the above components, and then bring the final volume up to 100 mL with DW.

Table S3. Recipe of dPTAP medium

Component	Stock Solution (mg/mL DW)	Quantity Used*
NH_4Cl	200	2.0 mL
$CaCl_2 \cdot 2H_2O$	100	0.5 mL
$MgCl_2 \cdot 6H_2O$	300	1.0 mL
KCl	140	1.0 mL
Hutner's trace elements	See the recipe in Table S1	1.0 mL
Acetic acid	-	1.0 mL
Tris (hydroxymethyl) aminomethane	-	2.42 g

* To prepare: make up to 1 liter with DW. Autoclave at 121°C for 20 minutes.

Movies S1-S10. 3D-representation movies corresponding to Figure 3. Color legends are as shown in Figure 3. Movies S1–S2: 3D-representation movies of a zero-control cell. Movie S3: 3D-representation movie of DBs in a zero-control cell. Movies S4–S5: 3D-representation movies of a starch cell. Movies S6: 3D-representation movie of DBs in a starch cell. Movies S7–S9: 3D-representation movies of a lipid cell. Movies S10: 3D-representation movie of DBs in the lipid cell.
